# Art as Healing

**DOI:** 10.1192/pb.bp.114.049544

**Published:** 2016-02

**Authors:** Alexandra Pitman

This book has a rather interesting back story. In 1946, a young man recently demobbed from the army took up a position as hospital artist at Netherne Hospital in south London. His only previous experience in such a role had been at a tuberculosis sanatorium, where the patients' goals were focused on mastering technique. In a psychiatric hospital 2 years before the birth of the National Health Service, his role seemed ill-defined and slightly overwhelming: not an occupational therapist, not an art teacher, but a hospital artist. Over time the patients' obvious enthusiasm and gratitude helped Edward Adamson crystallise an approach to his task that was non-patronising, respectful and unobtrusive, valued autonomy, and was probably very therapeutic. Arguing against the use of tranquillisers in favour of a solution originating from within, his view was that ‘art places the central responsibility for change upon the individual, rather than making him rely exclusively upon imposed treatment from outside’. Years later, he was founder chairman of the British Association of Art Therapists, and his pioneering work at Netherne is regarded as central to the emergence of art therapy.

This slim A4 book reads partly as an exhibition catalogue (the Adamson Collection hangs at Lambeth Hospital in London) and partly as an art therapy manual, setting out clear treatment maxims. ‘My own method is to be as passive as possible’, he explains. ‘I never attempt to interpret a person's work, particularly when he or she is painting. There is a great temptation to ascribe all sorts of psychological meanings to the paintings, quite independently of their originators. I do not even show a great deal of curiosity about the medical history of those who come to paint. It is so easy to prejudge people by labelling them’. Avoiding displaying patients' paintings on hospital walls in order to respect their privacy, Adamson invited selected visitors to a dedicated gallery, where he used the works to teach professionals how to understand and absorb the patient's point of view.

Adamson died in 1996, having spent the last years of his life in private practice in his Chelsea studio. *Art as Healing* was originally published in 1984, and was the UK charity Mind's book of the year in 1985. It has been reissued in 2014 in an unedited form, the only change being a preface to summarise Adamson's life's achievements. It is an enjoyable read, but the brief chapters are slightly disjointed, and I would have liked to have read an annotated version in which art therapists commented on the works or on Adamson's approach, adding their own contemporary examples. The chapters are categorised by theme, artist, and materials. Not all the works are accompanied by a clinical story but some spell out the distinct therapeutic advantage afforded to the patient in the act of its creation. It is not always clear if the labels represent the accounts of the artists or Adamson's own interpretation, and at times the role of a particular work in rerouting a patient's prognosis feels overplayed. The text also includes poems, but it is only apparent from the acknowledgements that these too are the work of patients, and it is not clear whether they are the work of one or more authors.

These are minor criticisms. Although the book feels a little dated in relation to its gendered language, the clinical material remains fresh. In the 1980s, as now, it reveals to psychiatrists how their patients view them, underlining the importance of empathy and intelligent kindness. Thirty years on, in the wake of the Matisse trial and its finding of no benefit of art therapy on negative symptoms or global functioning in schizophrenia, and in the context of draconian cuts in mental health services, it feels that a revised version would provide a fascinating account of how contemporary art therapists view the work of Adamson, setting the historical roots of art therapy in the context of an uncertain future. I would recommend this book to anyone interested in clinical stories, the history of asylums, techniques for communicating with disturbed patients, the Jungian approach to ‘letting things happen’ in art, the field of Outsider Art and what constitutes its boundaries, and individual-level interventions to challenge stigma. I would also suggest it to anyone who needs persuading that we all possess a creative energy that we may not have yet released.

